# Urodynamic Evaluation: Is It Useful for Vesicoureteral Reflux Management?

**DOI:** 10.3390/jcm14092883

**Published:** 2025-04-22

**Authors:** Joanna Bagińska-Chyży, Agata Korzeniecka-Kozerska

**Affiliations:** Department of Pediatrics and Nephrology, Medical University of Białystok, 17 Waszyngton Str., 15-274 Białystok, Poland; agatakozerska@poczta.onet.pl

**Keywords:** vesicoureteral reflux, urodynamics, pediatrics

## Abstract

**Background**: Vesicoureteral reflux (VUR) is a common childhood condition where urine flows backward from the bladder to the kidney, affecting 2–5 per 1000 live births. There is still no consensus on VUR diagnostic procedures, treatment options, or the most effective timing of treatment. This study aimed to identify factors influencing VUR management timing, with a focus on the role of urodynamic assessment. **Methods**: We retrospectively analyzed 100 children with VUR, splitting them into two groups: 50 who received urodynamic assessment and 50 who did not. The median age at diagnosis for both groups was 2 years (ranging from 1 month to 14 years). The children’s medical records were analyzed to determine gender, age at VUR diagnosis and its resolution, grade, and laterality. Additionally, we recorded the clinical history of bladder dysfunction, recurrent UTIs, and renal function parameters. For those who had urodynamic assessments, we also evaluated the age at initial testing and its findings. **Results**: Significant differences in treatment duration were found between the study groups: 1.17 years for the group after urodynamic assessment versus 2.83 years for the group without urodynamics (*p* < 0.001 *). The majority of patients assessed urodynamically had an overactive bladder (52%). **Conclusions**: Urodynamic assessment and effective treatment of bladder dysfunction accelerate VUR resolution.

## 1. Introduction

Vesicoureteral reflux (VUR) is characterized by the retrograde flow of urine from the bladder into the ureter, potentially reaching the kidney. It is among the most prevalent urologic conditions in childhood, with an estimated incidence of 2 to 5 per 1000 live births. Approximately one-third of children presenting with a urinary tract infection (UTI) are found to have VUR [1,2]. Recurrent UTIs due to VUR may lead to renal scarring, potentially resulting in hypertension, kidney insufficiency, or even kidney failure in severe cases. Historically, VUR was considered a congenital anomaly of the ureterovesical junction, a view that has been increasingly challenged. Recent research suggests that VUR pathogenesis is multifactorial, involving both primary structural abnormalities and secondary causes [3,4,5,6]. Bladder dysfunction plays a significant role, with conditions such as elevated detrusor pressures, overactive bladder, and neurogenic bladder–sphincter dyssynergia contributing to the retrograde flow of urine. Additionally, VUR is strongly influenced by genetic factors, as demonstrated by its tendency to run in families and the discovery of specific genetic variants linked to inherited susceptibility. Research has shown that VUR frequently runs in families, with siblings of affected children having a 30–50% likelihood of also being diagnosed with the condition. This risk is further elevated when both parents have a history of VUR, reinforcing the strong genetic component underlying its development [7,8]. Acquired factors, including recurrent urinary tract infections and chronic inflammation, may exacerbate reflux severity by altering bladder compliance and ureteral function [9]. Understanding VUR as a complex interplay of congenital, functional, genetic, and environmental factors is essential for optimizing diagnostic and therapeutic approaches.

The management of VUR in children involves a tailored approach based on the severity of the condition and the associated risk of renal damage. Observation and prophylactic antibiotic therapy are typically employed in mild cases, which often resolve spontaneously. For moderate to severe VUR, surgical interventions such as ureteral reimplantation or endoscopic injection may be indicated to prevent recurrent urinary tract infections (UTI) and progressive renal impairment [10,11]. Continuous monitoring through imaging studies and clinical follow-up is essential to evaluate treatment efficacy and to guide subsequent management decisions.

Numerous studies have demonstrated that bladder–bowel dysfunction (BBD) increases the risk of UTI, particularly in children with dilating VUR [11,12]. In such instances, urodynamic studies are considered critical in guiding the management of VUR. However, research linking urodynamic findings to the resolution of VUR remains limited. There is currently no consensus regarding the optimal diagnostic procedures, treatment strategies, or timing of intervention for VUR.

The objective of our study was to evaluate the impact of urodynamic studies in the diagnosis and treatment of patients with VUR. Specifically, we aimed to identify factors that may influence the duration of VUR management, with a particular focus on the role of urodynamic assessment.

## 2. Materials and Methods

**Figure 1:**
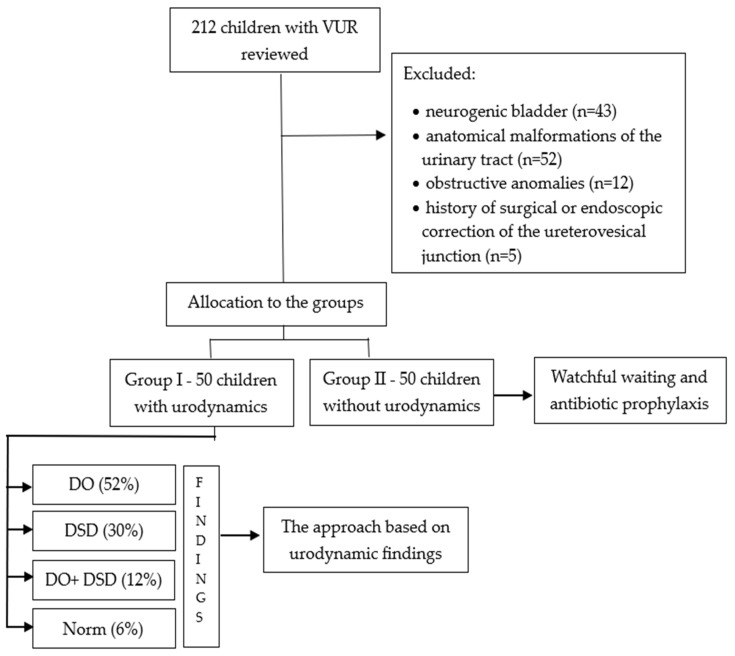
Flowchart diagram of VUR patient selection. DO—detrusor overactivity; DSD—detrusor–sphincter discoordination; VUR—vesicoureteral reflux.

This retrospective study analyzed 100 pediatric patients, with a median age of 2 years at the time of VUR diagnosis (ranging from 1 month to 14 years). The diagnosis of VUR was confirmed through voiding cystourethrography, and the severity of the condition was classified according to the International Reflux Study classification that categorizes VUR into five grades (I–V) based on the based on the voiding cystourethrography results as follows: grade I—VUR into a non-dilated ureter, grade II—into the ureter, pelvis, and calyces without dilation, grade III—with mild to moderate dilation of the ureter and renal pelvis, but without significant blunting of the calyces, grade IV—with moderate ureteral and renal pelvis dilation and some calyceal blunting, and grade V—severe dilation of the ureter, pelvis, and calyces [13]. The patients were stratified into two groups: group I included 50 children who underwent urodynamic assessment, while group II comprised 50 children who did not undergo urodynamic evaluation. In group I, the management of VUR was tailored to the urodynamic findings, whereas group II was managed with a conservative approach involving watchful waiting and antibiotic prophylaxis. Exclusion criteria for the study included neurogenic bladder dysfunction, anatomical malformations of the urinary tract other than VUR, posterior urethral valves and other obstructive anomalies, and a history of surgical or endoscopic correction of the ureterovesical junction. Details are shown in Figure 1.

Clinical data for all patients were systematically compiled using Microsoft Excel, based on their medical records. The analysis focused on demographic and clinical variables, including gender, age at the time of VUR diagnosis and resolution, VUR grade, laterality, and disease activity. Additionally, the clinical history of voiding dysfunctions, such as enuresis and daytime incontinence (DI), recurrent UTI, and renal function parameters—including serum creatinine levels and GFR at the time of VUR diagnosis and resolution—were documented. The GFR was calculated using the bedside Schwartz equation using serum creatinine and the patient’s height.

Urodynamic evaluation on each patient from group I was performed using the urodynamic device from Andromeda Medical Systems (Buckinghamshire, UK) placed in our department. The methods, definitions, and units for urodynamic investigation used in the study conform to those of the International Children’s Continence Society [14,15]. Data were collected on the age at which the assessment was conducted and the specific pharmacological treatments administered. Urodynamic diagnoses included detrusor overactivity (DO), detrusor–sphincter dysfunction (DSD), and normal bladder function.

Statistical analysis was performed using the commercial package Statistica 13.1 (StatSoft Inc, Tulsa, OK, USA). All studied parameters were analyzed using nonparametric tests: Mann–Whitney and ANOVA analysis. Correlations were assessed by the Spearman test. Values of *p* < 0.05 were considered significant.

The study protocol was approved by the Ethics Committee, Medical University of Bialystok, and was conducted in accordance with the Declaration of Helsinki.

## 3. Results

The characteristics of the patient cohort under study are detailed in Table 1. A substantial proportion, 79 out of 100 patients, was diagnosed with low-grade VUR classified as grade I or II. In contrast, the incidence of high-grade VUR (grades III or IV) was observed in 21 out of 100 patients. No patients were identified with VUR classified as grade V. Additionally, VUR was unilateral in 60% of the cases, with a twofold predominance of left-sided involvement, while bilateral VUR was present in 40% of the patients. The statistically significant difference was observed between the age of VUR diagnosis and its laterality. Bilateral VUR was diagnosed at a median age of 1.04 years (range: 0.25–8.6 years), compared to a median age of 3.07 years (range: 0.17–14 years) for unilateral VUR (*p* = 0.02; *p* < 0.05).

### 3.1. Gender

In both groups under study, girls constituted the majority of the enrolled patients. No statistically significant differences were observed in the studied parameters between girls and boys, with the exception of the presence of UTI (*p* = 0.03, *p* < 0.05). In our study, girls with VUR experienced UTIs more frequently than boys, with 54 out of 70 females (77%) having a history of UTIs, compared to 17 out of 30 males (57%).

### 3.2. Symptoms of VUR

Across the entire cohort, the most prevalent clinical manifestation of VUR was UTIs without concurrent voiding disorders, occurring in 74% of the cases. Voiding disorders were more commonly observed in group I, whereas UTIs were more frequently recorded in group II. Incontinence as the sole symptom of VUR was identified in only 8 out of 100 cases (8%). Both symptoms—UTI and voiding disorders—were concurrently present in 10 out of 100 cases (10%). Asymptomatic VUR was recorded in 8% of patients—6 in the group with urodynamic evaluation and 2 in the group without urodynamic assessment.

### 3.3. Urinary Tract Infections

Children from both study groups had a UTI history. Statistically significant differences were observed between patients with and without UTI concerning gender, clinical history of enuresis, age at VUR diagnosis, and its subsequent resolution (*p* = 0.03, *p* < 0.001, *p* = 0.02, *p* = 0.003, respectively; *p* < 0.05). Boys and children with a history of enuresis were less likely to have experienced UTIs. Additionally, in the group with a history of UTI, the age at VUR diagnosis was significantly lower, with a median of 1.5 years (ranging from 0.17 to 11 years), compared to 5.25 years (ranging from 0.42 to 14 years) in the group without a history of UTI.

### 3.4. Urodynamic Assessment

Among the 50 children who underwent urodynamic assessment, only 3 (6%) exhibited normal bladder function. The majority, comprising 26 out of 50 (52%), were diagnosed with DO. Additionally, 15 of the 50 children (30%) were identified with DSD. A subset of 6 children (12%) presented with both DO and DSD. A comparison between the grade of VUR and urodynamic findings revealed that children with lower VUR grades (I and II) exhibited a higher incidence of pathological urodynamic findings compared to those with higher VUR grades (III and IV), with a distribution of 72% versus 28%. Further details are provided in Table 2.

There were no statistically significant differences in the median age at which VUR was diagnosed between children who underwent urodynamic assessment and those who did not (*p* = 0.1). However, a statistically significant difference was observed in the duration of VUR treatment (<0.001; *p* < 0.05). In group I, where urodynamic studies were conducted and treatment was tailored based on the findings, VUR resolution occurred more rapidly compared to group II, which was managed with a watchful waiting strategy and antibiotic prophylaxis (1.17 years vs. 2.83 years).

Among the cohort of children who underwent urodynamic evaluation, 38% (19 out of 50) were younger than 5 years and were evaluated for suspected VUR following a UTI. Of these, a significant proportion—63% (12 out of 19)—were diagnosed with bilateral VUR classified as grade II in 5 cases, grade III in 6 cases, and grade IV in 1 case. In contrast, 62% (31 out of 50) of the patients in group I were older than 5 years and were toilet-trained. Among this older subgroup, 45% (14 out of 31) exhibited voiding disorders. Notably, children with enuresis were the oldest at the time of urodynamic assessment, with a median age of 9 years (range: 6.8–13.5 years), whereas those presenting with daytime incontinence had a median age of 5.5 years (range: 1–8 years). A detailed summary of the urodynamic findings stratified by age group is provided in Table 3.

## 4. Discussion

The traditional management of VUR, whether through surgical or medical means, was rooted in the historical view of primary VUR as a congenital anomaly of the ureterovesical junction. However, with the increasing use of urodynamic studies in children, this approach is being reconsidered. Emerging evidence has highlighted a strong association between VUR and BBD, with around 50% of children with VUR also exhibiting BBD. This percentage increases to 63% when urodynamic assessments are included [16,17,18,19]. BBD is a particularly influential factor influencing VUR resolution, especially in toilet-trained children. BBD is thought to cause elevated pressures during the storage and/or voiding phases, leading to intravesical anatomical changes that facilitate reflux. Therefore, assessing for BBD is crucial in the management of patients with VUR. The latest guidelines from the European Association of Urology/European Society of Paediatric Urology on managing VUR in children [10] recommend that the initial diagnostic process should involve a thorough inquiry into the presence of BBD. Urodynamic assessment is generally not included in the standard initial evaluation. However, video-urodynamic studies are recommended for patients suspected of having secondary VUR, including neurogenic bladder, or for children who do not respond to standard treatment for BBD. Since the 1990s, researchers have emphasized that while a voiding cystourethrogram is adequate for detecting VUR, accurate management requires a (video)urodynamic study. Scholtmeijer R.J. et al. [20] conducted a prospective study evaluating the utility of videourodynamics in managing 101 children with VUR. Among these, 39 were found to have bladder instability. The study demonstrated that treatment strategies were based on bladder function: children with bladder instability were treated with a combination of anticholinergic therapy and antibiotic prophylaxis, independent of VUR grade. In contrast, those with normal bladder function were managed according to reflux severity—either conservatively or through surgical intervention. The authors concluded that addressing the commonly associated bladder dysfunction often leads to the resolution of reflux in the majority of cases. It is important to recognize that video urodynamics can be costly and may require specialized equipment and trained professionals, making it less widely accessible. If video urodynamics is unavailable, we proved that traditional cystometry can still offer valuable insights. An additional advantage of urodynamic assessment is its ability to identify a potential neurogenic origin for BBD. Although this cause is relatively rare, it is frequently considered later in the diagnostic process, which may delay the establishment of an accurate diagnosis. By incorporating urodynamic testing into the management of VUR, there is an opportunity to expedite the identification of this underlying etiology. In our opinion, the urodynamic procedure, which some consider invasive due to the need for catheterization, carries less risk than the potential dangers of misdiagnosis and delaying the detection of coexisting voiding dysfunction, as well as postponing effective treatment. The most severe consequence of such delays is the progression to chronic kidney disease, which presents with a full spectrum of symptoms, including hypertension, proteinuria, and, ultimately, end-stage renal disease.

In our study, we found a very high incidence of bladder dysfunction (94%) among children after urodynamic assessment, with the highest prevalence of DO (52%). The majority of these cases involved girls with unilateral mild VUR. These findings are consistent with those reported in the most recent narrative review on the management of VUR in children [21], which highlights that VUR primarily affects two distinct groups: male infants with UTI or hydronephrosis and females who develop symptomatic VUR after the age of one. This distribution aligns closely with our observations. Our results also corroborate the findings of Batinic D. et al. [22], who conducted a urodynamic evaluation of 123 children aged 3 to 11 years with VUR and reported a high incidence of bladder dysfunction (75%), predominantly among girls. This gender distribution reflects the increased prevalence of VUR in females beyond the age of toilet training. Similar to their conclusions, we observed that children with mild VUR exhibited a higher prevalence of pathological urodynamic findings compared to those with higher VUR grades. The authors of that study identified DO as the most common urodynamic abnormality in children under 7 years old with lower unilateral VUR grades, in contrast to the presence of DSD in older children with higher bilateral VUR grades. In our study, DO was the most commonly observed urodynamic finding across the entire cohort, regardless of the patients’ age, VUR laterality, or severity.

Our analysis also revealed that enuresis was the factor that delayed the management and implementation of urodynamic assessment. This delay may be attributed to the ICCS recommendations [23], which suggest initiating active therapy for enuresis from the age of 6 years, typically without the need for invasive urodynamic studies. The management of enuresis follows a structured approach based on ICCS guidelines, beginning with an initial assessment to differentiate monosymptomatic nocturnal enuresis from non-monosymptomatic nocturnal enuresis, using voiding diaries and evaluating comorbidities such as constipation or sleep disorders. First-line therapy includes lifestyle modifications, such as fluid regulation and scheduled voiding, along with motivational therapy using positive reinforcement. Enuresis alarm therapy is recommended for children aged six and older, as it helps condition the child to wake at the onset of urination. If alarm therapy fails or a rapid response is needed, pharmacological treatment may be introduced, including desmopressin for nocturnal polyuria, anticholinergics for children with overactive bladder symptoms, and tricyclic antidepressants for severe refractory cases. Since invasive urodynamic studies are not part of standard enuresis management, the delay in urodynamic evaluation among enuretic children in our study likely reflects adherence to these guidelines, where conservative and pharmacological treatments are prioritized before considering further diagnostic assessments. Our study demonstrated that in children with VUR and voiding disorders, including enuresis, addressing underlying bladder dysfunction could be key to more effective VUR management in children.

What is more, in our study, a statistically significant difference was observed between the age of VUR diagnosis and its laterality. In our study, bilateral VUR was diagnosed in significantly younger patients compared to unilateral VUR. This difference may be attributed to the severity of symptoms associated with bilateral VUR. Patients with bilateral involvement are more likely to experience recurrent urinary tract infections, voiding dysfunction, and abnormalities detected on ultrasound, leading to earlier diagnosis. In contrast, mild unilateral VUR may present with less noticeable symptoms, potentially delaying its detection.

Our study has several limitations. First, it is a single-center study, which may limit the generalizability of the findings to other populations or clinical settings. Second, the retrospective nature of the study means that data collection was dependent on existing medical records, which may introduce potential biases or missing information. Additionally, we did not utilize video urodynamics, which could have provided more detailed anatomical and functional insights. Instead, we relied on traditional urodynamic assessments. Lastly, long-term follow-up of patients would be challenging due to the retrospective design, limiting our ability to assess the progression of bladder dysfunction over time. A notable strength of our research is the inclusion of a diverse cohort in the urodynamic assessment, encompassing not only toilet-trained children but also younger patients. Additionally, our study provides a comprehensive analysis of bladder dysfunction in VUR patients using standardized urodynamic assessments. Furthermore, the study contributes valuable clinical insights by examining the relationship between VUR characteristics and bladder dysfunction, which may help guide future diagnostic and management strategies.

## 5. Conclusions

Traditional management of VUR, which has long been based on the view of VUR as a congenital anomaly, is being reconsidered due to the increasing use of urodynamic studies in children. Post-urodynamic assessment has revealed that the most common bladder dysfunction associated with VUR is detrusor overactivity, particularly in girls with unilateral mild VUR. Additionally, BBD has emerged as a critical risk factor for VUR resolution, especially in toilet-trained children. Furthermore, voiding disorders, including enuresis, are highly prevalent among children with VUR and may play a key role in optimizing its management. These findings highlight the importance of assessing bladder function in VUR patients to guide more effective and individualized treatment strategies.

## Figures and Tables

**Table 1:** Characteristics of the studied cohort. * *p* < 0.05.

	All	Group I	Group II	Comparison
		*n* (%)		
Number of patients	100 (100)	50 (50)	50 (50)	
Boys/Girls	30 (30)/70 (70)	11 (22)/39 (78)	19 (38)/31 (62)	0.08
VUR diagnosis		Median (min—max)		
Age (years)	2 (0.17–14)	4 (0.17–10)	2.85 (0.33–14)	0.1
Weight (kg)	15 (5.25–50)	19.5 (6.8–50)	11 (5.25–50)	0.001 *
Height (cm)	100 (59–167)	118 (65–162)	84 (59–167)	<0.001 *
Creatinine (mg/dL)	0.35 (0.12–0.71)	0.36 (0.19–0.66)	0.32 (0.12–0.71)	0.12
GFR Schwartz (mL/min/1.73 m^2^)	120.3 (44–234)	123.5 (82–196)	109.7 (44–234)	0.15
VUR resolution				
Age (years)	6 (1.5–17)	8 (1.5–17)	5 (2.33–17)	0.02 *
Weight (kg)	22 (10–69)	26 (10–68)	19 (12.5–69)	0.02 *
Height (cm)	118 (78–174)	129.5 (78–174)	110 (90–170)	0.01 *
Creatinine (mg/dL)	0.4 (0.19–0.82)	0.44 (0.19–0.74)	0.37 (0.2–0.82)	0.07
GFR Schwartz (mL/min/1.73 m^2^)	123.1 (52.4–219.6)	122.1 (71.6–180.4)	123.9 (52.4–219.6)	0.83
Duration of treatment (years)	2 (0.33–15)	1.17 (0.33–4)	2.84 (0.83–7.5)	<0.001 *
VUR grade		*n (%)*		
1	18 (18)	7 (14)	11 (22)	0.3
2	61 (61)	29 (58)	32 (64)	0.54
3	18 (18)	12 (24)	6 (12)	0.12
4	3 (3)	2 (4)	1 (2)	0.56
VUR laterality				
Right/Left	20 (20)/40 (40)	24 (48)/7 (14)	16 (32)/13 (26)	0.69/0.14
Bilateral	40 (40)	19 (38)	21 (42)	0.27
Incontinence				
Enuresis	8 (8)	6 (12)	2 (4)	0.14
DI	4 (4)	3 (6)	1 (2)	0.31
Enuresis and DI	6 (6)	6 (12)	0 (0)	0.01 *
No incontinence	82 (82)	35 (70)	47 (94)	0.001 *
UTI history	84 (84)	39 (78)	45 (90)	0.29

**Table 2:** Relation of VUR grade and urodynamic findings.

Urodynamic Findings	VUR Grade				VUR Laterality	
	I	II	III	IV	Unilateral	Bilateral
DO	5	12	8	1	15	11
DSD	1	13	1	0	8	7
DO + DSD	1	2	2	1	2	4
Normal bladder function	0	2	1	0	1	2
Total	7	29	12	2	26	24

DO—detrusor overactivity; DSD—detrusor–sphincter discoordination; VUR—vesicoureteral reflux.

**Table 3:** Relation of age and urodynamic findings.

Urodynamic Findings	Age Groups	
	<5 y.o	>5 y.o
DO	10	16
DSD	7	8
DO + DSD	0	6
Normal bladder function	2	1
Total	19	31

DO—detrusor overactivity; DSD—detrusor–sphincter discoordination.

## Data Availability

The data presented in this study are available on request from the corresponding author. The data are not publicly available for ethical and privacy reasons.
